# Controllable Growth of the Graphene from Millimeter-Sized Monolayer to Multilayer on Cu by Chemical Vapor Deposition

**DOI:** 10.1186/s11671-015-1164-0

**Published:** 2015-11-26

**Authors:** Jinyang Liu, Zhigao Huang, Fachun Lai, Limei Lin, Yangyang Xu, Chuandong Zuo, Weifeng Zheng, Yan Qu

**Affiliations:** College of Physics and Energy, Fujian Normal University, Fuzhou, 350117 People’s Republic China; Fujian Provincial Key Laboratory of Quantum Manipulation and New Energy Materials, Fuzhou, 350117 People’s Republic China

**Keywords:** Graphene, Chemical vapor deposition, Stacking order, Oxide nanoparticle, Hydrogen concentration, 68.65.-k, 81.15.Gh, 78.30.-j

## Abstract

As is well established, mastery to precise control of the layer number, stacking order of graphene, and the size of single-crystal monolayer graphene is very important for both fundamental interest and practical applications. In this report, millimeter-sized single-crystal monolayer graphene has been synthesized to multilayer graphene on Cu by chemical vapor deposition. The relationship of the growth process between monolayer graphene and multilayer graphene is investigated carefully. Besides the general multilayer graphene with Bernal stacking order, parts of multilayer graphene with non-Bernal stacking order were modulated under optimized growth conditions. The oxide nanoparticle on the Cu surface derived from annealing has been found to play the key role in nucleation. In addition, the hydrogen concentration impacts significantly on the layer number and shape of the graphene. Moreover, a possible mechanism was proposed to understand the growth process discussed above, which may provide an instruction to graphene growth on Cu by chemical vapor deposition.

**Electronic supplementary material:**

The online version of this article (doi:10.1186/s11671-015-1164-0) contains supplementary material, which is available to authorized users.

## Background

Graphene, a monolayer of carbon atom arranged in a two-dimensional hexagonal lattice, has attracted increasing attention due to its novel properties [1, 2] and the promising applications for diverse fields [3, 4]. To date, various methods have been explored to prepare graphene, such as mechanical cleavage [5], chemical method [6], epitaxy on SiC [7, 8], and chemical vapor deposition (CVD) [9, 10] on metal foil. Among these methods, the CVD method has aroused great attention due to its potential to be a procedure to produce graphene with high quality in a large scale. In fact, the graphene with a 30in. growth on Cu foil by CVD has been successfully synthesized [11]. However, the obtained graphene were polycrystalline structures with a high density of grain boundaries and defects, which should be reduced or entirely eliminated because they impede carrier transport [12, 13] by intervalley scattering [14, 15], mechanically weaken the graphene [16, 17], and promote undesirable surface reactions with adsorbates from the environment [18]. To overcome these disadvantages, large-sized and high-quality single-crystal monolayer graphene is on the agenda, and recently, a synthesis of monolayer graphene with the lateral size reaching 1 cm was reported [19].

However, in the CVD growth process, single-crystal monolayer graphene always accompanies multilayer graphene [20]. What is the growth mechanism of the multilayer graphene? What is the relationship of the growth mechanism between monolayer graphene and multilayer graphene? Up to now, two mechanisms have been widely recognized in multilayer graphene growth. One is called on-top growth mechanism [21, 22] based on the diffusion-limited growth on Cu. In this process, most of such carbon species may be captured by the first layer and contribute to the growth of the first layer. Only a small percentage of the carbon species is able to go across the edge of the first layer to reach the second layer. Another growth mechanism is called underlayer growth mechanism [23]. Specifically, the first layer graphene grows on the Cu surface and is considered as a template for the growth of the second layer. Some active species can penetrate a graphene overlayer, which leads to carbon intercalation and growth of the second graphene layer. However, the relationship between the growth mechanism of monolayer graphene and multilayer graphene is still ambiguous and needs further investigation.

In this report, we report an approach to synthesize large-sized single-crystal monolayer graphene and multilayer graphene with different stacking orders on Cu by CVD. By controlling the growth parameters, millimeter-sized single-crystal monolayer graphene grew on Cu by CVD. Furthermore, multilayer graphene with Bernal stacking order and non-Bernal stacking order was also synthesized under optimized growth conditions. In addition, the relationship of the growth process between monolayer graphene and multilayer graphene is investigated carefully. The oxide nanoparticle on the Cu surface derived from annealing was found to play an important role in nucleation, while the hydrogen concentration impacted greatly on the layer number and shape of the graphene. Finally, a possible mechanism was proposed to reveal the growth process, which may advance our understanding on the growth of the large-sized single-crystal monolayer graphene and multilayer graphene with different stacking order.

## Methods

### Graphene Growth

The synthesis of graphene was carried out in a split tube furnace using CVD. The typical process to synthesize large-sized single-crystal monolayer graphene is shown as follows. The Cu foils (25 μm thick, 99.8 % polycrystalline, Alfa Aesar #13382) used as substrate were cut into a small rectangle shape with a size of 2.5 cm. The Cu foil was then etched in dilute hydrochloric acid and cleaned by acetone under ultrasonic and then dried by flowing N_2_ gas. Next, the Cu foil was placed in a quartz tube in the furnace and heated to 1080 °C with flowing 300 sccm Ar, and then the temperature was held for 4 h with flowing 300 sccm Ar and 50 sccm H_2_. Graphene growth was carried out by starting the CH_4_ flow at 0.5 and 50 sccm H_2_ with 3 h in the same tube furnace following completion of the annealing/reduction step of the Cu foil. The sample was cooled down quickly to room temperature by opening the furnace under 300 sccm Ar and 4 sccm H_2_ after growth. The process to grow multilayer graphene is similar to the method shown above. The main difference are shown as follows: the annealing time is 3 h in the annealing/reduction step, the CH_4_ and H_2_ flow is 0.5 and 25 sccm, respectively, with 0.5 h in the growth process. The method to transfer graphene grown on copper foils is similar to the previous report [24].

### Characterization

The Raman spectra were recorded at room temperature using HORIBA Jobin Yvon Evolution with laser excitation at 532 nm with power less than 5 mW. The optical microscopy was characterized with the Olympus BX51M in reflection mode at room temperature. The scanning electron microscopy (SEM) was characterized by Hitachi SU-8010. The element analysis was recorded by energy-dispersive X-ray spectroscopy (EDS, AMETEK) attached on SEM. Transmission electron microscopy (TEM) attached with selected area electron diffraction (SAED) was characterized by JEOL JEM-2010 TEM.

## Results and Discussion

### Large-Sized Single-Crystal Monolayer Graphene

**Fig. 1:**
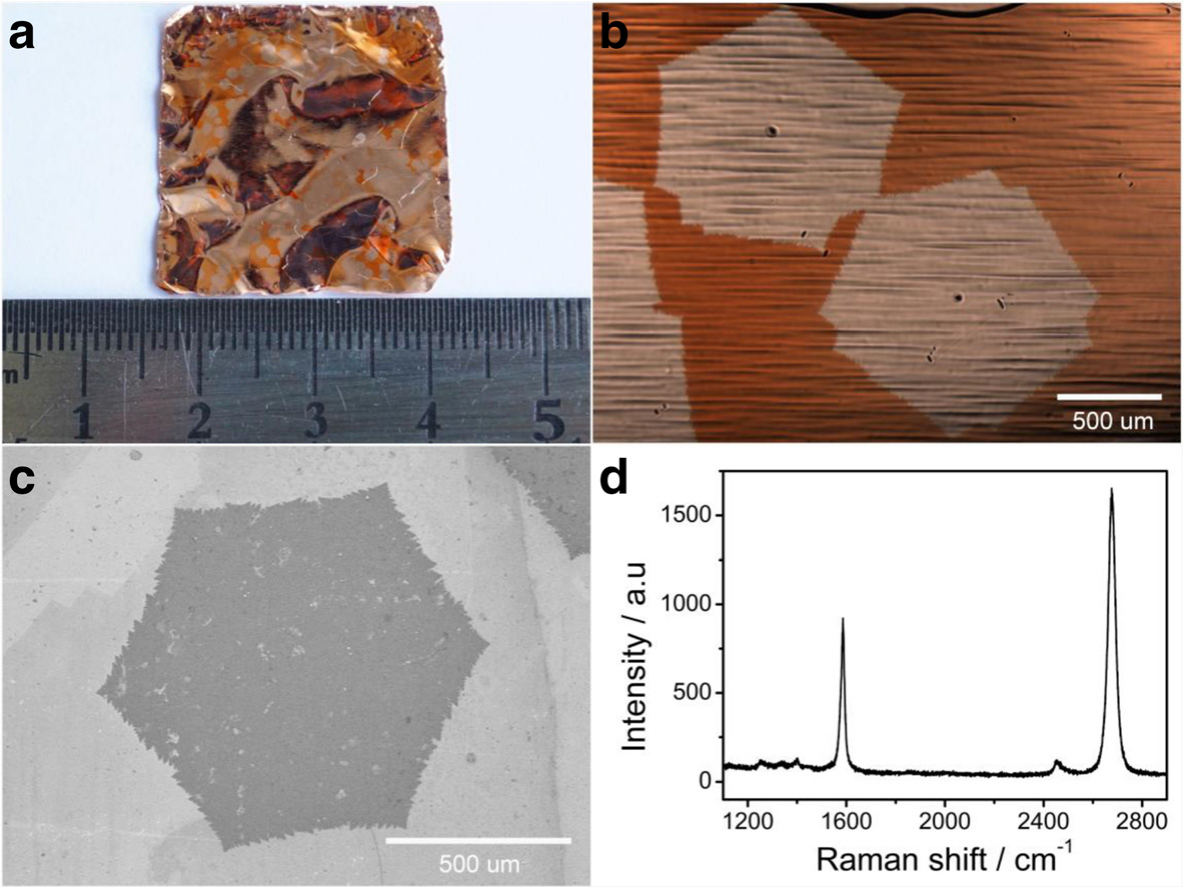
**a** The photograph of the as-grown graphene domains on Cu foil after oxidation. **b** Optical microscopy images of the graphene domains in (**a**). **c**, **d** The SEM image and Raman spectroscopy of the single crystal graphene transferred to SiO_2_, respectively

In general, the treatment of the Cu foil is found to be a critical step to grow high-quality graphene. At present, various methods have been developed to grow large-sized single-crystal monolayer graphene, for instance, suppressing evaporative loss of Cu [10], preannealing Cu at atmospheric pressure [25], melting and resolidifying of Cu [26], and using a Cu enclosure [9] or circumfluent CVD method [27]. In our experiment, the Cu foil was cleaned with dilute hydrochloric acid and acetone under ultrasonic to obtain the fresh and native Cu surface. Then virgin Cu foil was annealed at 1080 °C in hydrogen and argon gas to further eliminate the sharp wrinkles, steps, and defects [28]. After annealing with a long time, some oxide nanoparticles derived from the mild oxidation residual were formed as previous reports [28, 29]. The oxide nanoparticle acted as the nucleation site, which not only reduced the nucleation barrier energy but also controlled the density of the graphene domains. Then, 0.5 sccm CH_4_ and 50 sccm H_2_ were introduced to synthesize a single-crystal monolayer graphene. A series of experiments were carried out, and the typical results are shown in Fig. 1. The Cu foil with grown graphene was oxidized firstly by heating at 200 °C for 1 min in air to make the graphene domains optically visible [30]. From Fig. 1a, it can be seen that the density of the graphene domains is fairly low and the graphene domains are in the size of millimeters. The optical microscopy shown in Fig. 1b demonstrates that the graphene domains were in the size of about 1.2 mm with hexagonal shape. Moreover, a nanoparticle in the middle of the graphene domain can be observed clearly, and this phenomenon appeared in most of graphene domains.

SEM was employed to further reveal the structure of the samples, before which the graphene domains on Cu foil were transferred to the SiO_2_/Si substrate (the thickness of the SiO_2_ with thermal oxidized was about 300 nm) [24]. Figure 1c shows the SEM image of the graphene domains transferred on the representative SiO_2_/Si substrate; it can be seen that the graphene domains are uniform with the size of 1.2 mm except some impurities produced in the transferred process. As is well acknowledged, the Raman spectroscopy is an excellent approach to evaluate the quality, thickness, and uniformity of the graphene [31, 32]. Therefore, the Raman spectroscopy of the graphene domains transferred on the SiO_2_/Si substrate was performed and the typical result is shown in Fig. 1d. The G-peak and 2D-peak are observed clearly and located at ~1589.2 and ~2682.7 cm^−1^, respectively. Meanwhile, the I_2D_/I_G_ is about 1.80 and the 2D band exhibits a single Lorentzian curve with a full width at half maximum (FWHM) of ~32.3 cm^−1^, confirming that it is a single-layer graphene. On the other hand, the intensity of the disorder-induced D-peak (~1345 cm^−1^) is very weak, indicating the high quality of the graphene. To further confirm the crystallinity of the graphene domains, selected area electron diffraction (SAED) patterns were performed and the results are shown in Additional file 1: Figure S1. It can be seen that only one set of hexagonal diffraction spots without rotation was observed in four arbitrary probed sites, indicating it is a single crystalline. From the results shown above, the large-sized single-crystal monolayer graphene domains can be able to grow on Cu by CVD.

### Multilayer Graphene with Bernal and non-Bernal Stacking Order

**Fig. 2:**
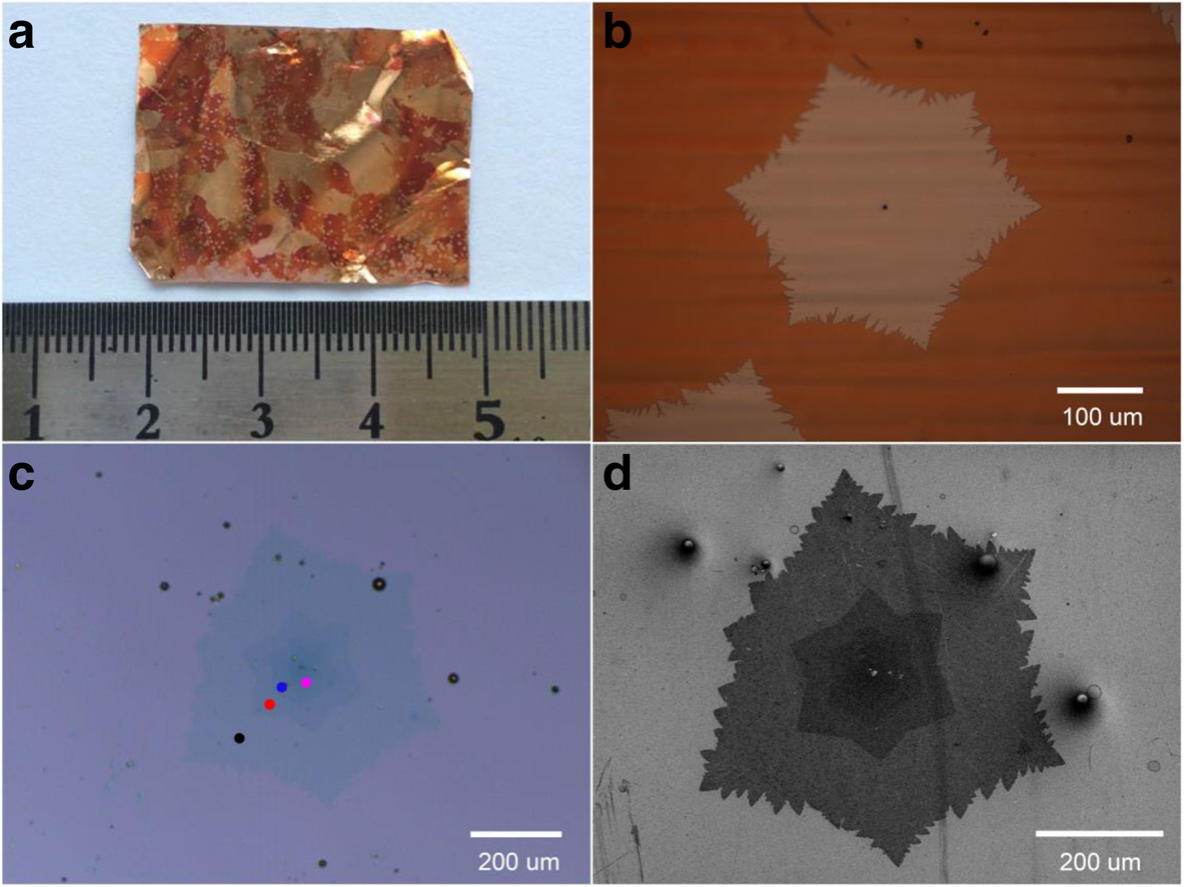
**a** The photograph of the as-grown graphene domains on Cu foil after oxidation. **b** Optical microscopy images of the graphene domains in (**a**). **c**, **d** The optical microscopy and scanning electron microscopy of the multilayer graphene domains transferred to SiO_2_, respectively

Besides the large-sized single-crystal monolayer graphene, another interesting phenomenon is found when the hydrogen concentration decreases in the growth process. A series of experiments with hydrogen concentration from high to low in the growth process are performed, and the typical results are shown in Additional file 1: Figure S2. A small multilayer graphene in the center region appeared when the hydrogen concentration decreased to 38 sccm, while the size of the multilayer graphene increased with the hydrogen concentration further down to 29 sccm. When the hydrogen concentration further reduced to 25 sccm, a beautiful multilayer graphene was obtained and the results are shown in Fig. 2. Figure 2a shows the photograph of the Cu foil used to synthesize graphene after oxidation; the graphene domains can be observed easily but with a relatively low density. To observe the graphene domain more clearly, the samples were further characterized by the optical microscope, and the results shown in Fig. 2b elucidate that the graphene domains have a hexagonal shape with some jagged edges. It should be pointed out that a nanoparticle can be clearly identified in the middle of the graphene domain, and these phenomena are observed in nearly all graphene domains.

To further characterize the sample, optical microscopy has been conducted and the results are shown in Fig. 2c. The graphene domains are hexagonal in shape with high contrast. In the outer regions, it exhibits a single-layer structure; toward the center, the graphene layer number increases from single layer, bilayer, to multilayer. The shape and the direction of the graphene crystal are almost similar. It should be noted that the interfaces between these graphene layers are constructed naturally, which is interesting in scientific research, both in theory and in experiment. Then, the samples were further characterized by SEM, and the corresponding SEM image was presented in Fig. 2d. It can be seen that the graphene domains are multilayer in structure which was constructed with single layer, bilayer, and multilayer from the outer regions to the center. The results of SEM image are consistent with the optical microscopy shown in Fig. 2c. Furthermore, the nanoparticle in the middle of the multilayer graphene can also be observed clearly.

**Fig. 3:**
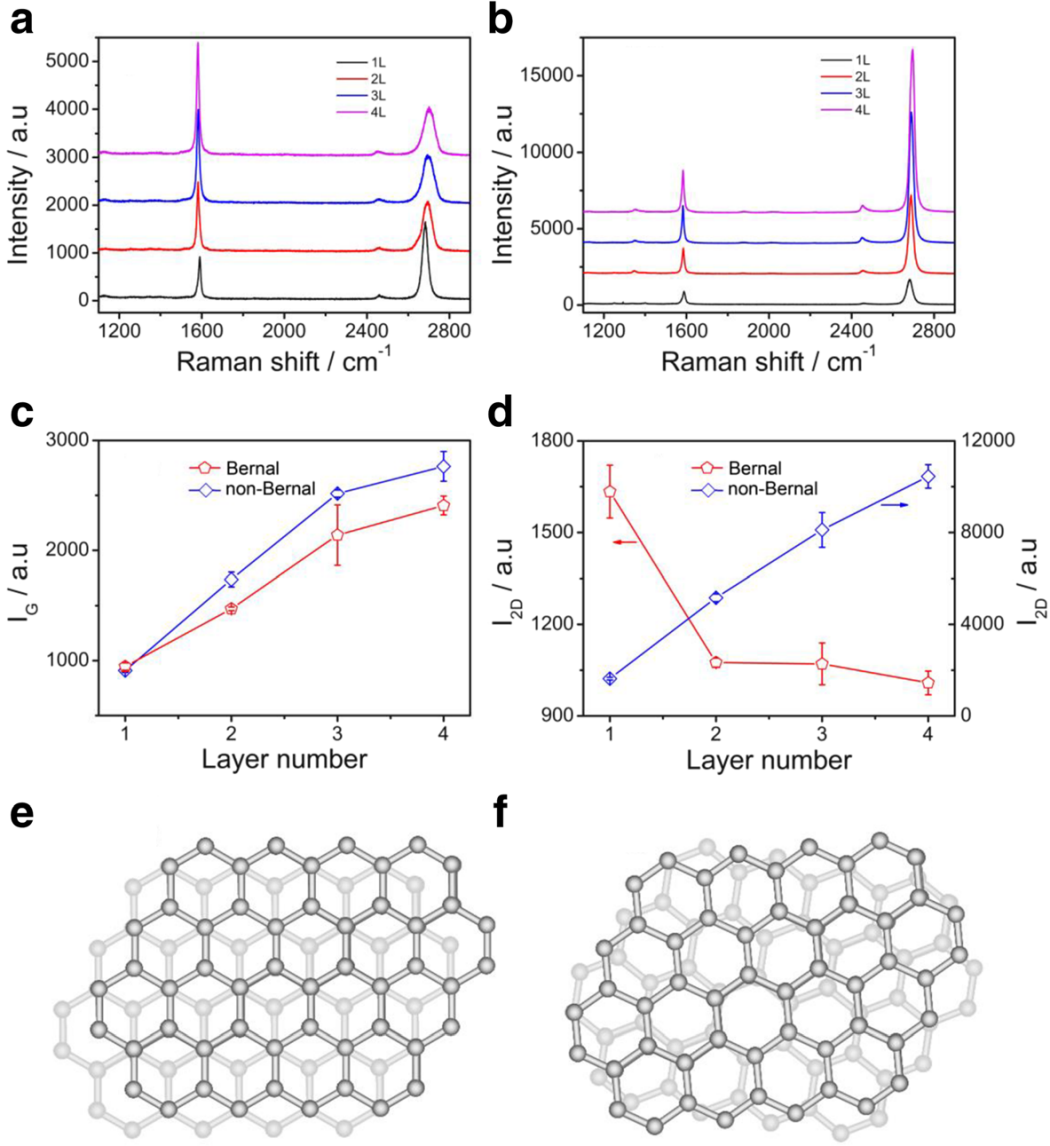
**a**, **b** The Raman spectroscopy of the multilayer graphene with Bernal and non-Bernal stacking order, respectively. **c**, **d** The intensity of the D and 2D peak shown in (**a**) and (**b**) as a function of the graphene layer number. **e**, **f** The scheme of the multilayer graphene with Bernal and non-Bernal stacking order, respectively

Then, the multilayer graphene transferred on the SiO_2_/Si substrate was characterized by Raman spectroscopy, and the typical results are displayed in Fig. 3a with the probed position shown in Fig. 2c. The Raman spectrum demonstrates that the G-peak and 2D-peak are observed clearly and located at ~1582 and ~2690 cm^−1^, respectively, with a weak disorder-induced D-peak located at ~1345 cm^−1^, indicating the high quality of the multilayer graphene. To identify more details, the 2D band has been deconvoluted by Lorentzian function. Additional file 1: Figure S3 (shown in supplementary information) reveals that the black circle area shown in Fig. 2c shows the I_2D_/I_G_ value of ~1.73, and the 2D band exhibits a single Lorentzian curve with the full width at half maximum (FWHM) of ~28.66 cm^−1^, corroborating its single-layer structure. The red circle area shown in Fig. 2c exhibits the I_2D_/I_G_ value of ~0.73, and the 2D band exhibits four fitted Lorentzian curves with the FWHM of ~24.57 cm^−1^, indicating it is a bilayer graphene. The blue circle area shown in Fig. 2c shows the I_2D_/I_G_ value of ~0.50, and the 2D band exhibits six fitted Lorentzian curves with the FWHM of ~22.56 cm^−1^, indicating it is a trilayer graphene. And the pink circle area is a tetralayer structure. From single-layer graphene to tetralayer graphene, the positions of the G band are 1589.3, 1582.8, 1582.3, and 1581.8 cm^−1^, respectively, decreasing as the layer number increases as shown in Additional file 1: Figure S6(a); however, the positions of the 2D band are 2681.3, 2695.9, 2696.8, and 2704.1 cm^−1^, respectively, increasing as the layer number increases as shown in Additional file 1: Figure S6(b). On the other hand, from single-layer graphene to tetralayer graphene, the intensity of the G band linearly increases as shown in Fig. 3c, while the 2D band decreases from single-layer to bilayer and almost stable to trilayer and tetralayer as shown in Fig. 3d. From the results shown above, the 2D peak of bilayer graphene can be fitted with four Lorentzian curves, while the 2D peak in trilayer graphene can be fitted with six Lorentzian curves, and the line shape of the peak shows little asymmetry with no obvious shoulder, which suggests that the multilayer graphene have Bernal (ABA) stacking order rather than rhombohedral (ABC) stacking order [33]. Furthermore, the tetralayer graphene’s 2D peak can be fitted with three Lorentzian curves and its symmetrical line shape reveals its Bernal (ABAB) stacking signature (shown in Additional file 1: Figure S3(d)). Therefore, it can be concluded that the multilayer graphene domains shown above have Bernal stacking order [32, 33], which is the general crystal structure in graphene (shown in Fig. 3e).

Surprisingly, besides the Bernal stacking order of the multilayer graphene shown above, some multilayer graphene with another stacking order are observed. The optical microscopy images of the typical sample transferred to the SiO_2_/Si substrate are shown in Additional file 1: Figure S4. The results of the Raman spectra are shown in Fig. 3b with the probed position shown in Additional file 1: Figure S4. The main features are all similar, specially, the G-peak and 2D-peak are observed clearly, while the disorder-induced D-peak (~1345 cm^−1^) is weak, indicating that the quality of the multilayer graphene is very high. From the outer to inner position, the Raman shift of G band are 1589.4, 1585.6, 1583.2, and 1583.2 cm^−1^ (shown in Additional file 1: Figure S6(a)), respectively, decreasing toward the center. On the other hand, the intensity of G-peak increases in a linear manner from the outer to inner regions (shown in Fig. 3c), indicating that the layer number increases due to more carbon atoms contributing to this vibration mode. In the outer position, it can be seen that the I_2D_/I_G_ is ~1.79, and the 2D band exhibits a single Lorentzian curve (shown in Additional file 1: Figure S5) with a FWHM of ~29.32 cm^−1^, confirming it is a single-layer graphene. The Raman shift of 2D band from the outer to inner regions in the multilayer graphene are 2682.5, 2688.7, 2688.6, and 2694.9 cm^−1^ (shown in Additional file 1: Figure S6(b)), respectively, increasing as the layer number rises, while the intensity increases from 1631.4 to 11045.3 a.u. as shown in Fig. 3d. Furthermore, in order to further understand the Raman spectra of the graphene domains with different layers, the deconvolution of the 2D band with Lorentzian function were carried out, and the results are shown in Additional file 1: Figure S5 . It can be seen that the 2D band were all well fitted with one Lorentzian component. To further understand this phenomenon, the I_2D_/I_G_ of the graphene in probed positions from the outer (monolayer graphene) to inner regions were collected and are shown in Additional file 1: Figure S7; it can be seen that the I_2D_/I_G_ value increases from 1.79, 2.97, 3.23, to 3.79. And the intensity of 2D band increases linearly with a slope of 3229.3 as shown in Fig. 3d. All these features shown above are similar to the single-layer graphene; but why? A possible explanation is that the stacking order between the graphene layers is arbitrary and consequently, the coupling effect between graphene layers is low. Therefore, one can conclude that the multilayer graphene shown above is a non-Bernal stacking order (shown in Fig. 3f), which is consistent with the previous results [34].

Therefore, as the hydrogen concentration decreases in the growth process, the multilayer graphene domains could be obtained and the size of the multilayer graphene in the center region increases. Most of the multilayer graphene have a Bernal stacking order; however, parts of multilayer graphene have non-Bernal stacking order. This phenomenon is interesting because precise control of the layer number and stacking order of graphene are very important to both fundamental interest and practical applications.

### Growth Mechanism

**Fig. 4:**
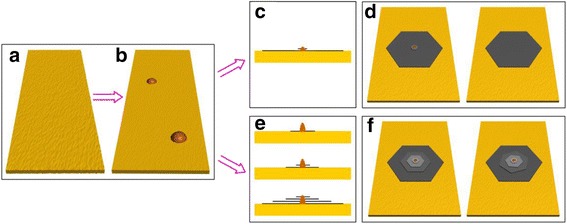
The Scheme depicts the proposed mechanism for graphene domain growth. **a** The smooth Cu foil was obtained by cleaning with dilute hydrochloric acid and acetone under ultrasonic. **b** Formation of large oxide nanoparticles resulting from the mild oxidation by trace amounts of oxygen in Ar gas on the CVD chamber. **c**, **d** The large single-crystal monolayer graphene resulting from nucleation on the oxide nanoparticle with the growth speed on bottom layer graphene is high while on the top layer, graphene is suppressed in the high hydrogen concentration condition. **e**, **f** The multilayer graphene resulting from nucleation on the oxide nanoparticle with the growth speed between the bottom layer and top layer graphene is relatively equal in the low hydrogen concentration condition

From the experiments shown above, large single-crystal monolayer graphene and multilayer graphene with different stacking order could be synthesized on Cu by CVD under optimized growth conditions. However, how can we understand the phenomenon shown above? What is the relationship of the growth mechanism between monolayer graphene and multilayer graphene? Given the findings in our experiments and the facts in previous reports [21, 27, 35], a possible mechanism based on on-top growth process was proposed which is shown in Fig. 4. In this mechanism, two steps were essential. One is the Cu foil annealing at the high temperature, it not only reduces the impurity and eliminates the sharp wrinkles, steps, and defects effectively, but also produces some oxide nanoparticles [28, 29] from the mild oxidation residual. In order to determine the element of the nanoparticle, the EDS were carried out. Additional file 1: Figure S8 shows the typical EDS spectrum of the probe sites on the nanoparticle and not on the nanoparticle; it can be seen that the O signals are observed clearly on the nanoparticle comparing with those not on the nanoparticle besides the Cu and C signals, indicating that the nanoparticle may be an oxide of copper. The oxide nanoparticle acts as the nucleation site, which not only reduces the nucleation barrier energy but also controls the density of the graphene domains as shown in Fig. 4a and b. This phenomenon is consistent with the results of the optical microscopy and SEM image in Fig. 1 or Fig. 2 where a nanoparticle was observed in the middle of the graphene domain either monolayer or multilayer. In addition, the surface oxygen on the Cu surface may have existed as indicated by the EDS of the nanoparticle. The graphene nucleates on the oxide nanoparticle and begins to grow tuned from edge-attachment-limited growth to the diffusion (mass transport)-limited growth due to the surface oxygen that existed [19]. Consequently, the edge of the graphene contacted on the Cu surface is jagged, which is consistent with the results shown in Fig. 1 or Fig. 2 and the previous reports [19]. The hydrogen concentration is another key point in the growth process. The hydrogen concentration not only controls the layer number of the graphene domains, but also affects the shape of the graphene domains. In the growth process, the subsequent graphene layer continues nucleating on the oxide nanoparticle and keeps on growing with the template of the bottom graphene by absorbing active carbon, and consequently, a multilayer graphene nucleation is formed. However, the growth speed of the top layer graphene is relatively low due to loss of contact with the catalytic substrate and affected by the hydrogen concentration heavily. In the condition of high hydrogen concentration, the growth speed on the bottom layer graphene is much higher due to more active carbon catalyzed by the Cu surface, while the top layer graphene nucleated on the oxide nanoparticle would be suppressed or even disappear due to the high hydrogen concentration at high temperature, and therefore, the large-sized single-crystal monolayer graphene can be obtained as shown in Fig. 4c and d. The corresponding experiment results are shown in Fig. 1. With low hydrogen concentration, the growth speed between the bottom layer and top layer graphene is relatively equal, so, multilayer graphene is obtained as displayed in Fig. 4e and f. To further confirm the mechanism proposed above, the experiments that the multilayer graphene growth with increasing time were carried out, and the typical results are shown in Additional file 1: Figure S9. When the growth time increases from 10, 20, to 40 min, the size of the graphene contacted the Cu surface (first layer) increases from 63.9, 128.7, to 170.1 μm, while the size of the subsequent graphene (second layer) increases from 7.1, 8.4, to 8.9 μm. The results indicate that the growth speed in the first layer is much larger than the second layer, which is consistent with the on-top mechanism. The graphene layer contacted to the Cu surface would grow fast, while the subsequent layers would grow very slowly due to loss of contact with the catalytic substrate. On the other hand, the stacking order between different graphene layers may be influenced by the fluctuation of the temperature, carbon source, and so forth in the growth process. However, the precise factor which induced the different stacking order is still unknown and needs further exploration in the next step.

## Conclusions

Large-sized single-crystal monolayer graphene has been synthesized to multilayer graphene with Bernal stacking order and non-Bernal stacking order on Cu by CVD under optimized growth conditions. The oxide nanoparticle derived from the mild oxidation residual on Cu surface plays an important role in nucleation and controls the density of the graphene domains. While the hydrogen concentration impacts greatly on the shape and layer number of the graphene. The relationship of the growth process between monolayer graphene and multilayer graphene is investigated carefully. Furthermore, a possible mechanism based on on-top growth mechanism was proposed to understand the growth process, which may have a great significance on the growth of graphene domains with a different size, layer number, and stacking order.

## Additional file

Additional file 1:
**Supplementary information.** Figure S1. (a) The TEM image shows the corner of the graphene domains. (b–e) Selected area electron diffraction (SAED) data for small regions indicated 1 to 4. These SAED data confirm the single-crystalline structure of the graphene domains as they have the same set of sixfold symmetric diffraction points. Figure S2. The optical microscopy images of the multilayer graphene with increasing size in the center region grown by decreasing hydrogen concentration and keeping the methane for constant (0.5 sccm CH_4_). (a) 38 sccm H_2_; (b) 29 sccm H_2_. Figure S3. The deconvolution of the 2D band of the (a) monolayer, (b) bilayer, (c) trilayer, and (d) tetralayer graphene with Lorentzians function as shown in Fig. 3a. Figure S4. The optical microscopy images of the multilayer graphene with non-Bernal stacking transferred to SiO_2_. Figure S5. The deconvolution of the 2D band of the (a) monolayer, (b) bilayer, (c) trilayer, and (d) tetralayer graphene with Lorentzians function as shown in Fig. 3b. Figure S6. The G (a) and 2D (b) peak position of the multilayer grahene with Bernal and non-Bernal stacking order as shown in Fig. 3a and b, respectively. Figure S7. The I_2D_/I_G_ value of the multilayer graphene with Bernal and non-Bernal stacking order as shown in Fig. 3a and b, respectively. Figure S8. The typical EDS spectrum of the probe site on the nanoparticle and not on the nanoparticle. Figure S9. The optical microscopy images of the multilayer graphene growth with 32 sccm H_2_, 0.5 CH_4_ at different time. (a) 10 min, (b) 20 min, (c) 40 min. (DOC 6452 kb)

## Abbreviations

CVD: chemical vapor deposition
EDS: energy-dispersive X-ray spectroscopy
FWHM: full width at the half maximum
SAED: selected area electron diffraction
SEM: scanning electron microscopy
TEM: transmission electron microscopy
