# Supplementary material for: Controllable Growth of the Graphene from Millimeter-Sized Monolayer to Multilayer on Cu by Chemical Vapor Deposition
Source: Nanoscale Res Lett. 2015 Nov 26;10:455. doi: 10.1186/s11671-015-1164-0 (PMC4661165; doi:10.1186/s11671-015-1164-0)
Supplement: Additional file 1: — Supplementary information. Figure S1. (a) The TEM image shows the corner of the graphene domains. (b–e) Selected area electron diffraction (SAED) data for small regions indicated 1 to 4. These SAED data confirm the single-crystalline structure of the graphene domains as they have the same set of sixfold symmetric diffraction points. Figure S2. The optical microscopy images of the multilayer graphene with increasing size in the center region grown by decreasing hydrogen concentration and keeping the methane for constant (0.5 sccm CH4). (a) 38 sccm H2; (b) 29 sccm H2. Figure S3. The deconvolution of the 2D band of the (a) monolayer, (b) bilayer, (c) trilayer, and (d) tetralayer graphene with Lorentzians function as shown in Fig. 3a. Figure S4. The optical microscopy images of the multilayer graphene with non-Bernal stacking transferred to SiO2. Figure S5. The deconvolution of the 2D band of the (a) monolayer, (b) bilayer, (c) trilayer, and (d) tetralayer graphene with Lorentzians function as shown in Fig. 3b. Figure S6. The G (a) and 2D (b) peak position of the multilayer grahene with Bernal and non-Bernal stacking order as shown in Fig. 3a and b, respectively. Figure S7. The I2D/IG value of the multilayer graphene with Bernal and non-Bernal stacking order as shown in Fig. 3a and b, respectively. Figure S8. The typical EDS spectrum of the probe site on the nanoparticle and not on the nanoparticle. Figure S9. The optical microscopy images of the multilayer graphene growth with 32 sccm H2, 0.5 CH4 at different time. (a) 10 min, (b) 20 min, (c) 40 min. (DOC 6452 kb) [file 11671_2015_1164_MOESM1_ESM.doc]

**Supplementary Information to “Controllable Growth of the Graphene from Millimeter-sized Monolayer to Multilayer on Cu by Chemical Vapor Deposition**”

Jinyang Liu*^a,b*^*, Zhigao Huang*^a,b^*, Fachun Lai*^a,b^*, Limei Lin *^a,b^*, Yangyang Xu *^a^*, Chuandong Zuo *^a^*, Weifeng Zheng *^a^*, Yan Qu *^a^*

*^a^*College of Physics and Energy, Fujian Normal University, Fuzhou, 350117, P. R. China

*^b^*Fujian Provincial Key Laboratory of Quantum Manipulation and New Energy Materials, Fuzhou, 350117, P. R. China

*Corresponding Author. E-mail: jyliu@fjnu.edu.cn; TEL: +86-18120795008


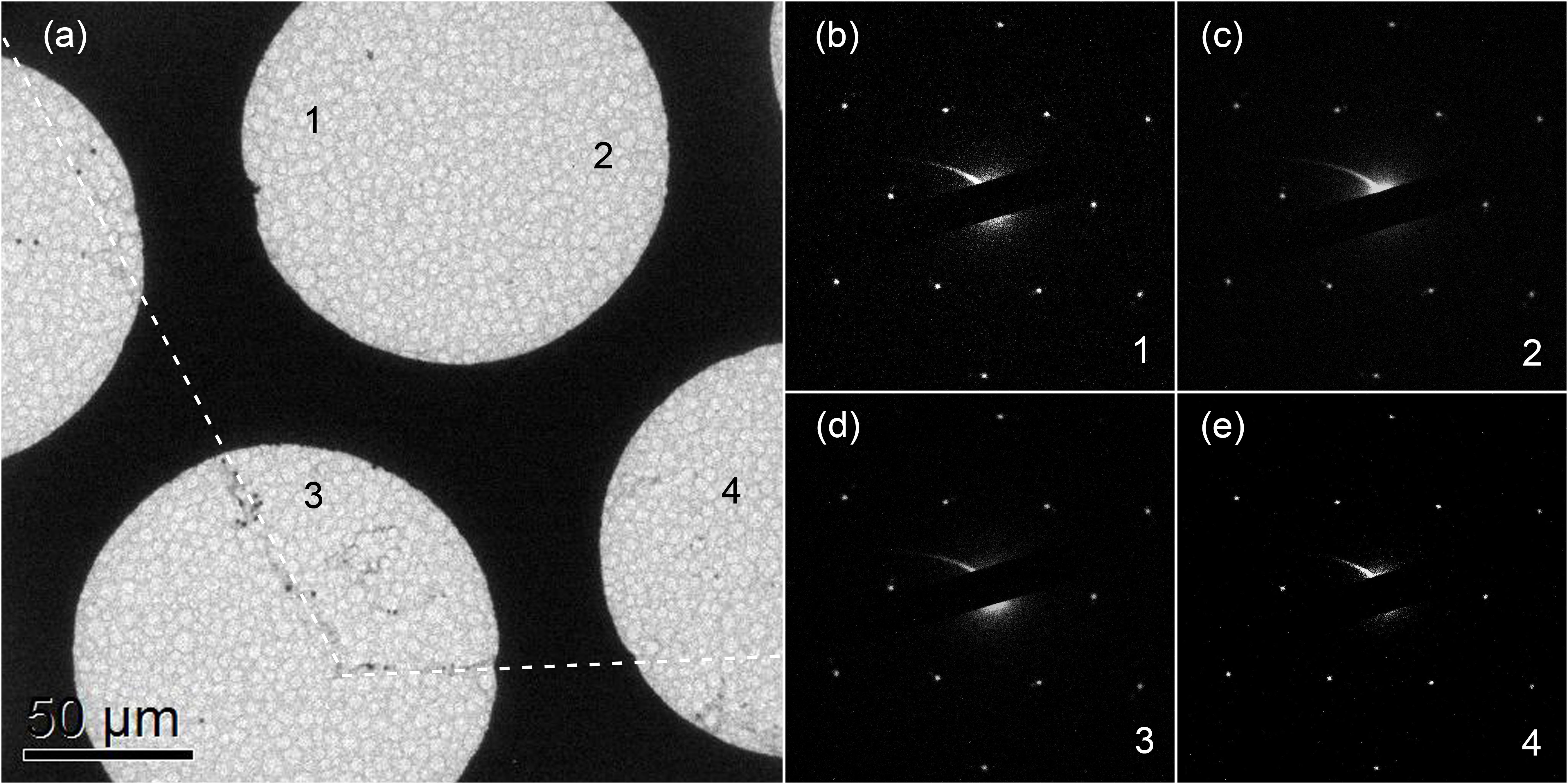


Fig. S1 (a) The TEM image shows the corner of the graphene domains. (b–e) Selected area electron diffraction (SEAD) data for small regions indicated 1 to 4. These SAED data confirm the single-crystalline structure of the graphene domains as they have the same set of six-fold symmetric diffraction points.


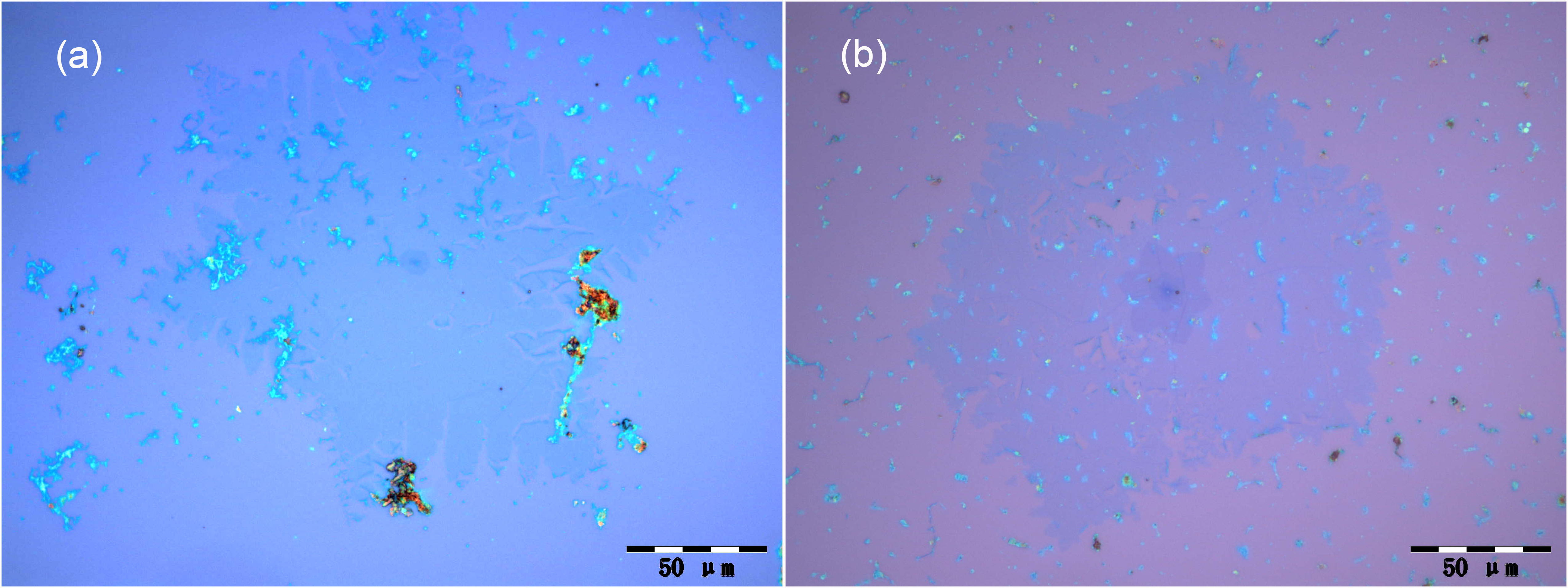


Fig. S2 The optical microscopy images of the multilayer graphene with increasing size in the centre region grown by decreasing hydrogen concentration and keeping the methane for constant (0.5 sccm CH_4_). (a) 38 sccm H_2_; (b) 29 sccm H_2_.


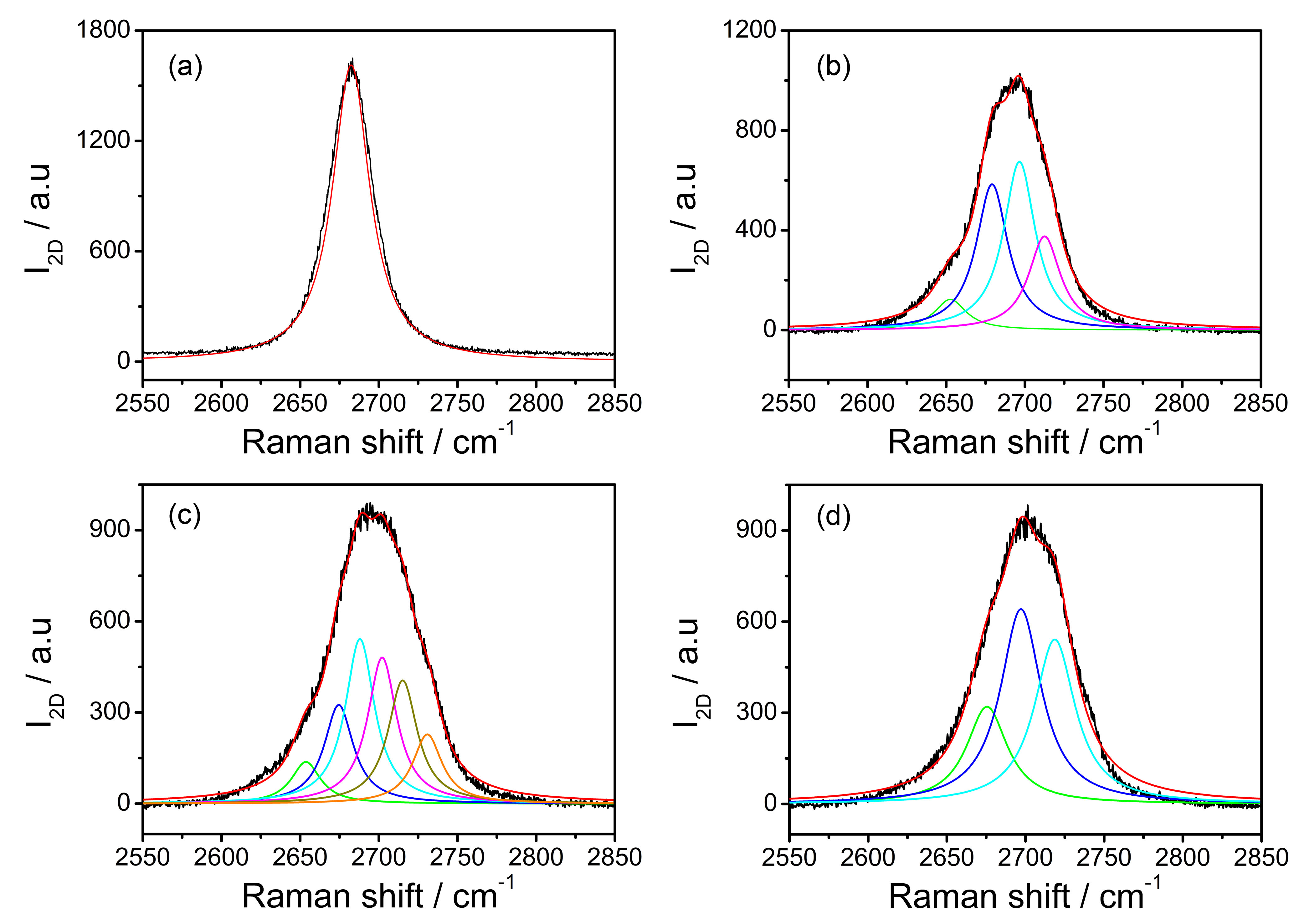
 Fig. S3. The deconvolution of the 2D band of the (a) monolayer, (b) bilayer, (c) trilayer and (d) tetralayer graphene with Lorentzians function as shown in Fig. 3(a).


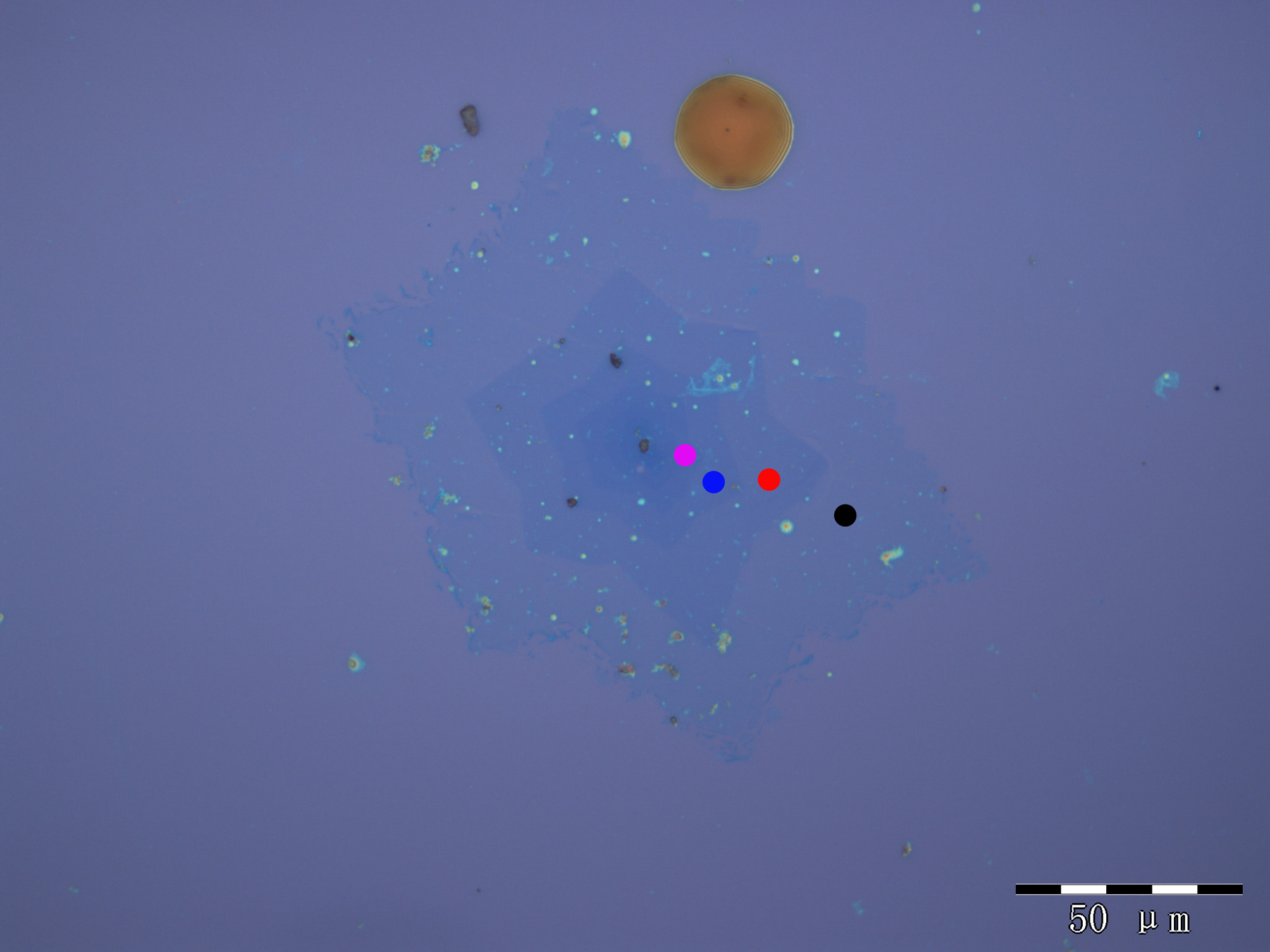


Fig. S4. The optical microscopy images of the multilayer graphene with non-Bernal stacking transferred to SiO_2_.


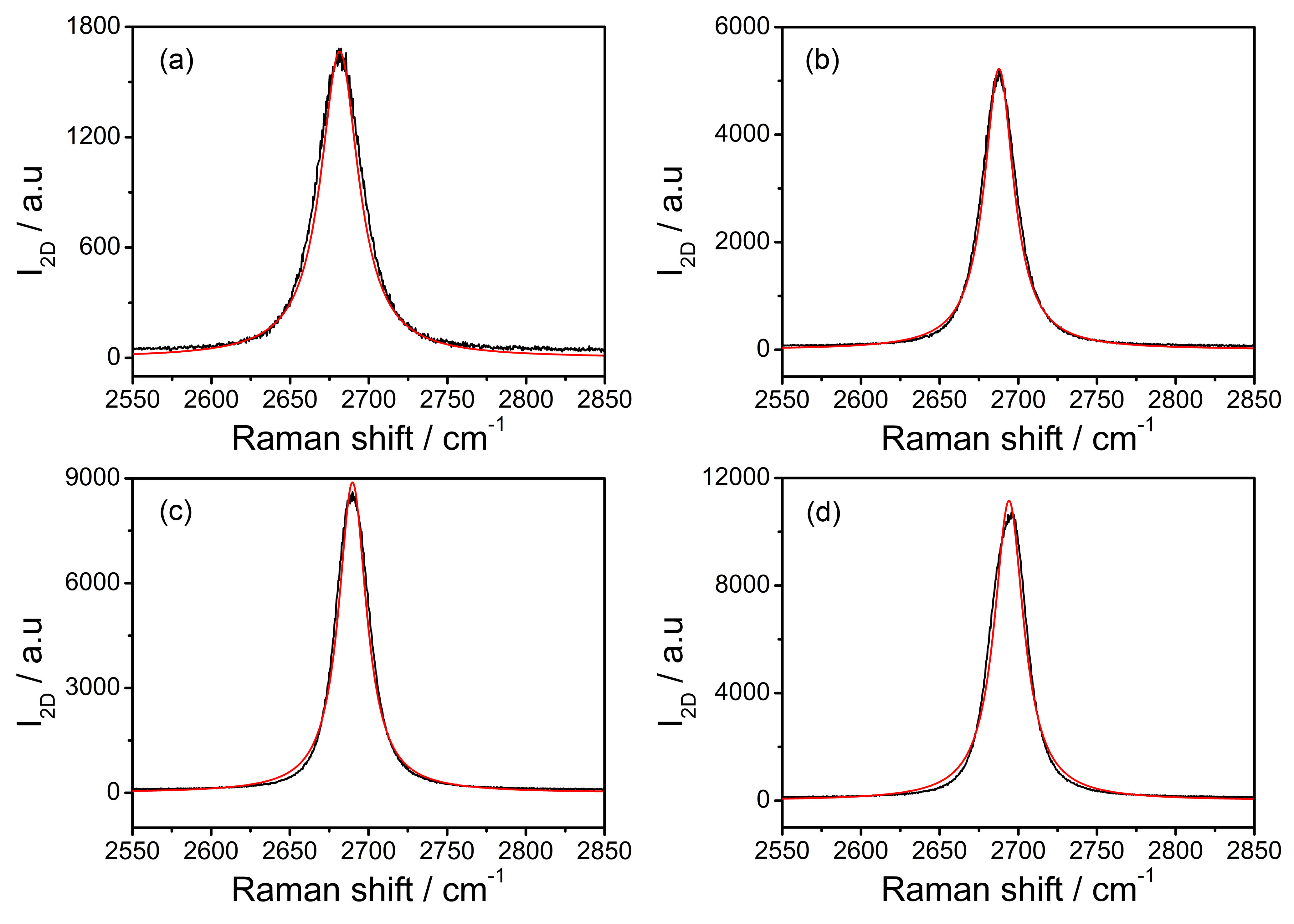


Fig. S5. The deconvolution of the 2D band of the (a) monolayer, (b) bilayer, (c) trilayer and (d) tetralayer graphene with Lorentzians function as shown in Fig. 3(b).


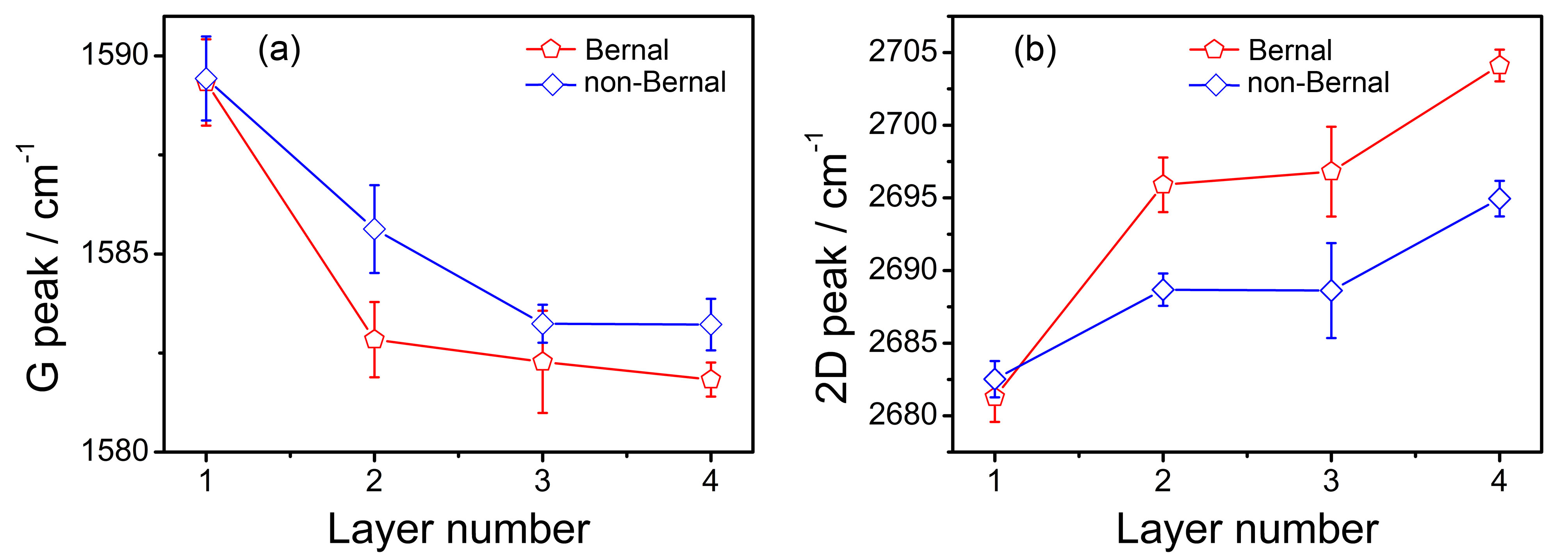


Fig. S6. The G (a) and 2D (b) peak position of the multilayer grahene with Bernal and non-Bernal stacking order as shown in Fig. 3 (a) and (b) respectively.


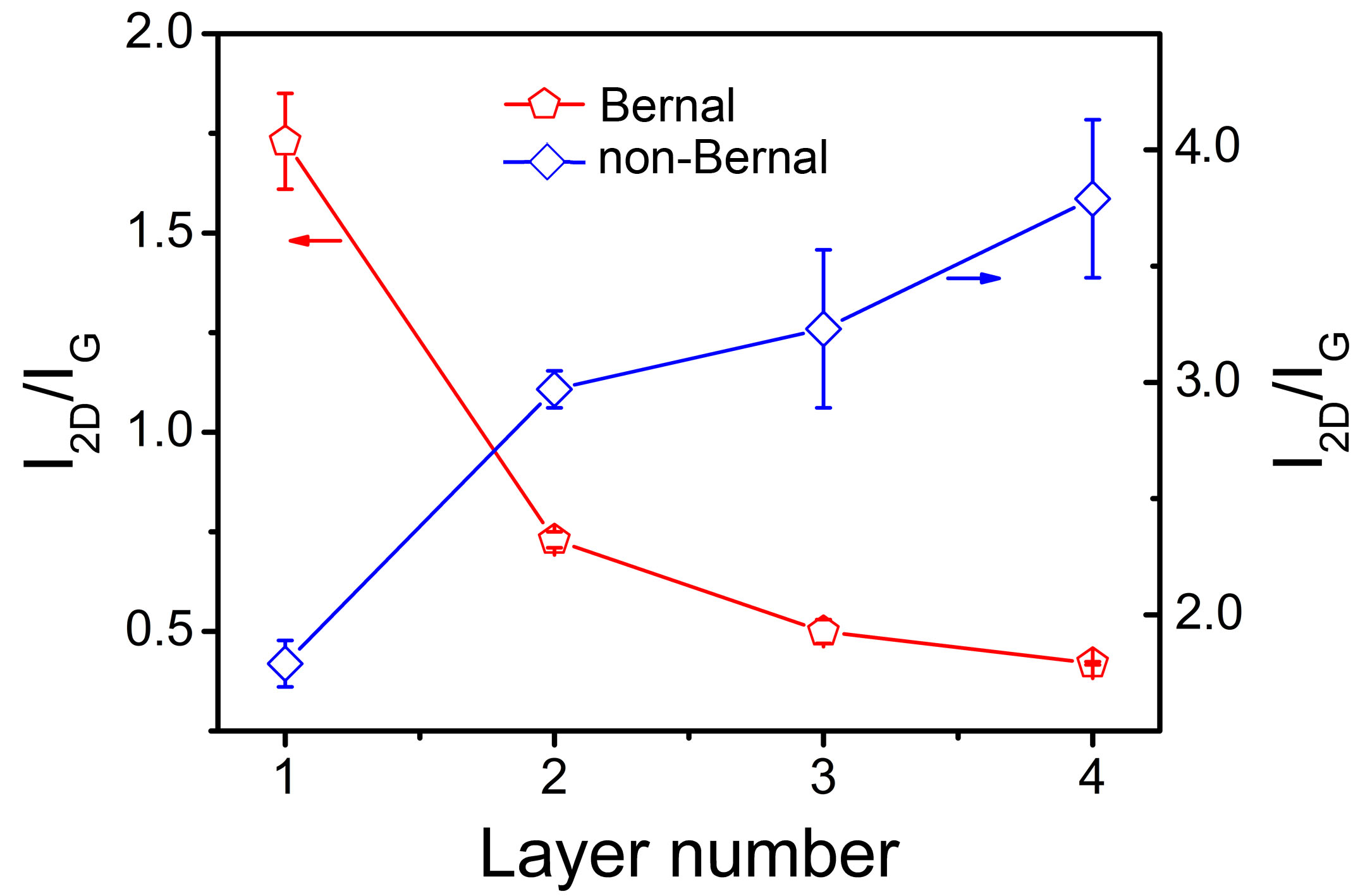


Fig. S7 The I_2D_/I_G_ value of the multilayer graphene with Bernal and non-Bernal stacking order as shown in Fig. 3 (a) and (b) respectively.


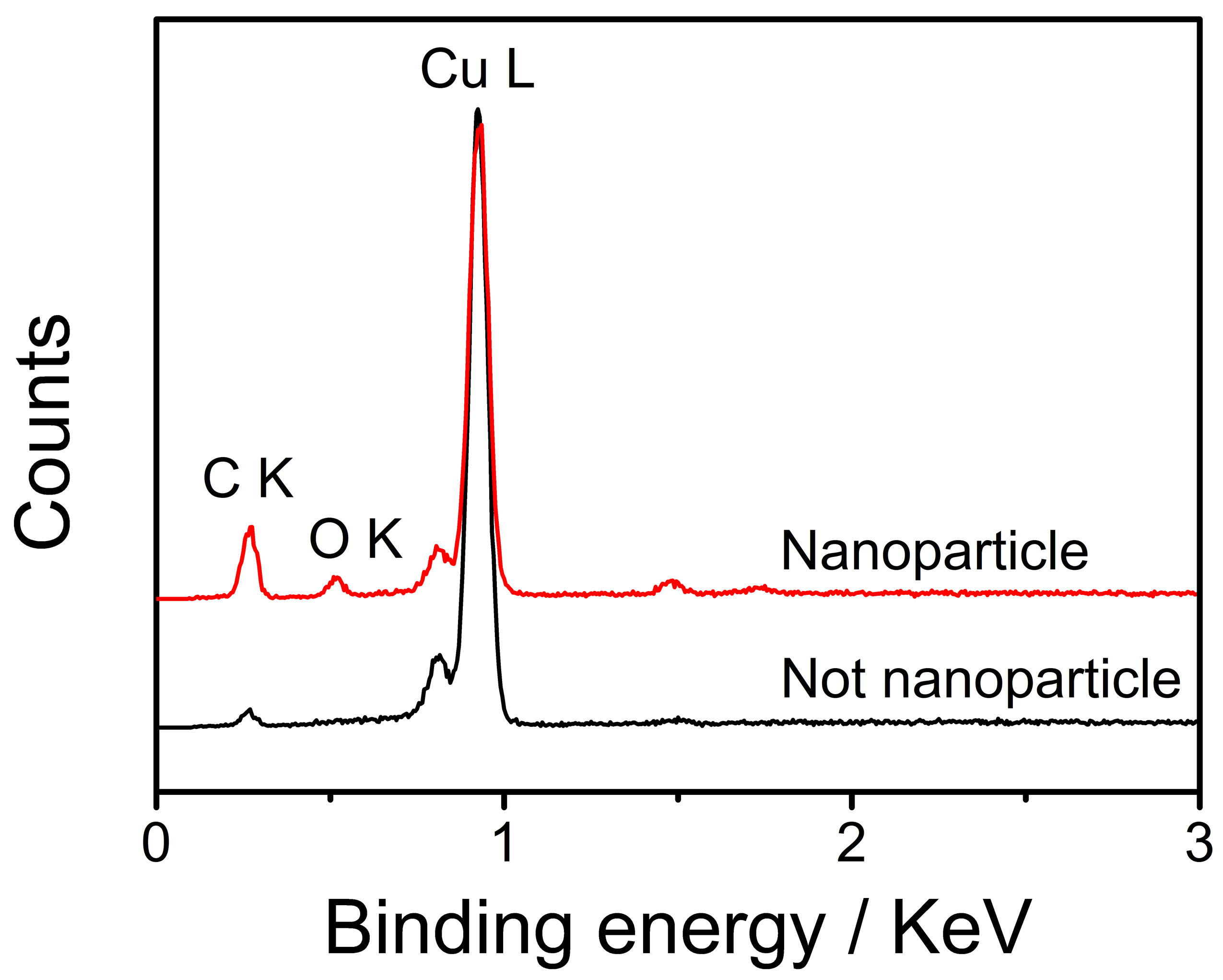


Fig. S8 The typical EDS spectrum of the probe site on the nanoparticle and not on the nanoparticle.


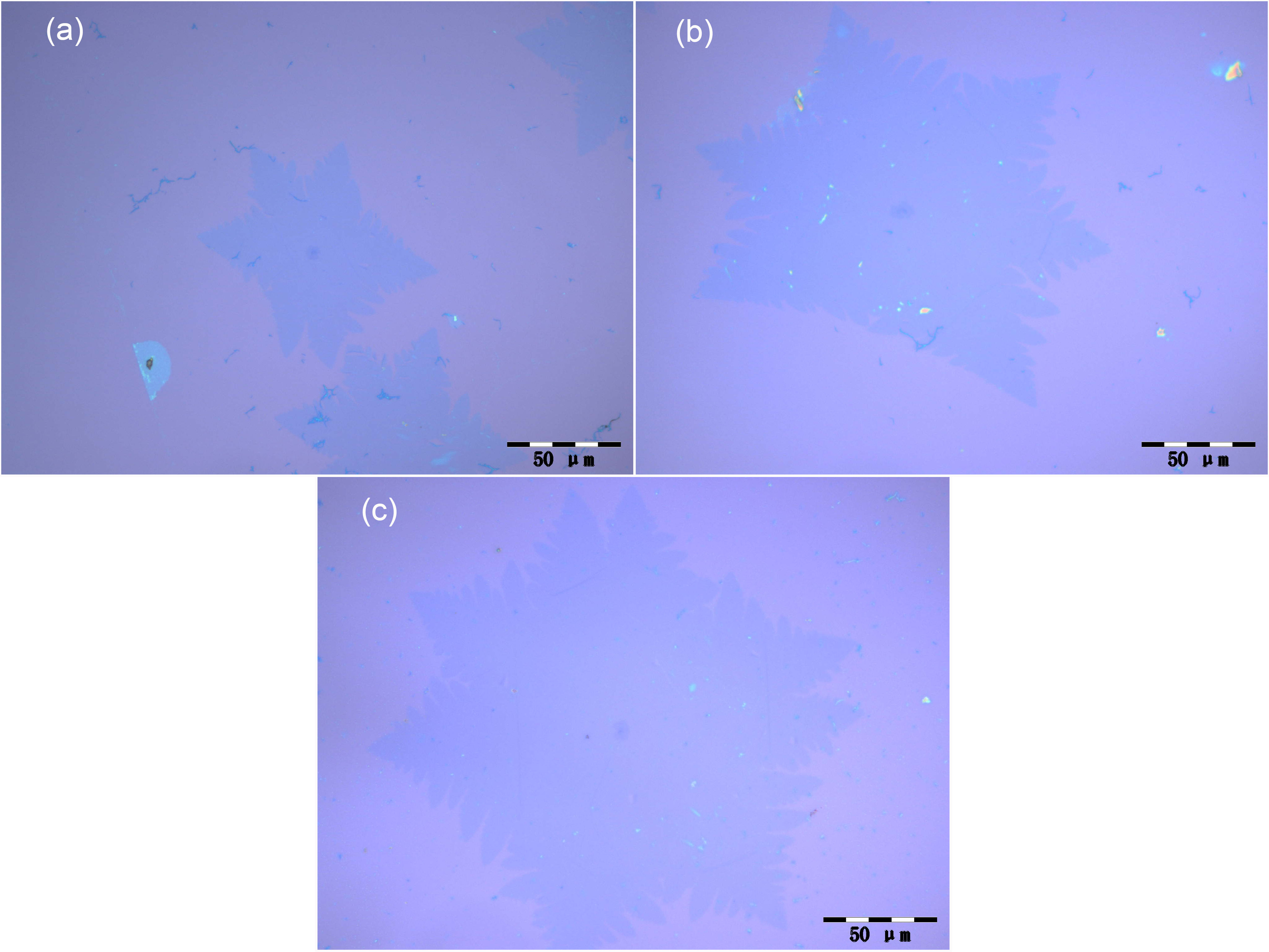


Fig. S9 The optical microscopy images of the multilayer graphene growth with 32 sccm H_2_, 0.5 CH_4_ at different time. (a) 10min, (b) 20min, (c) 40min.
